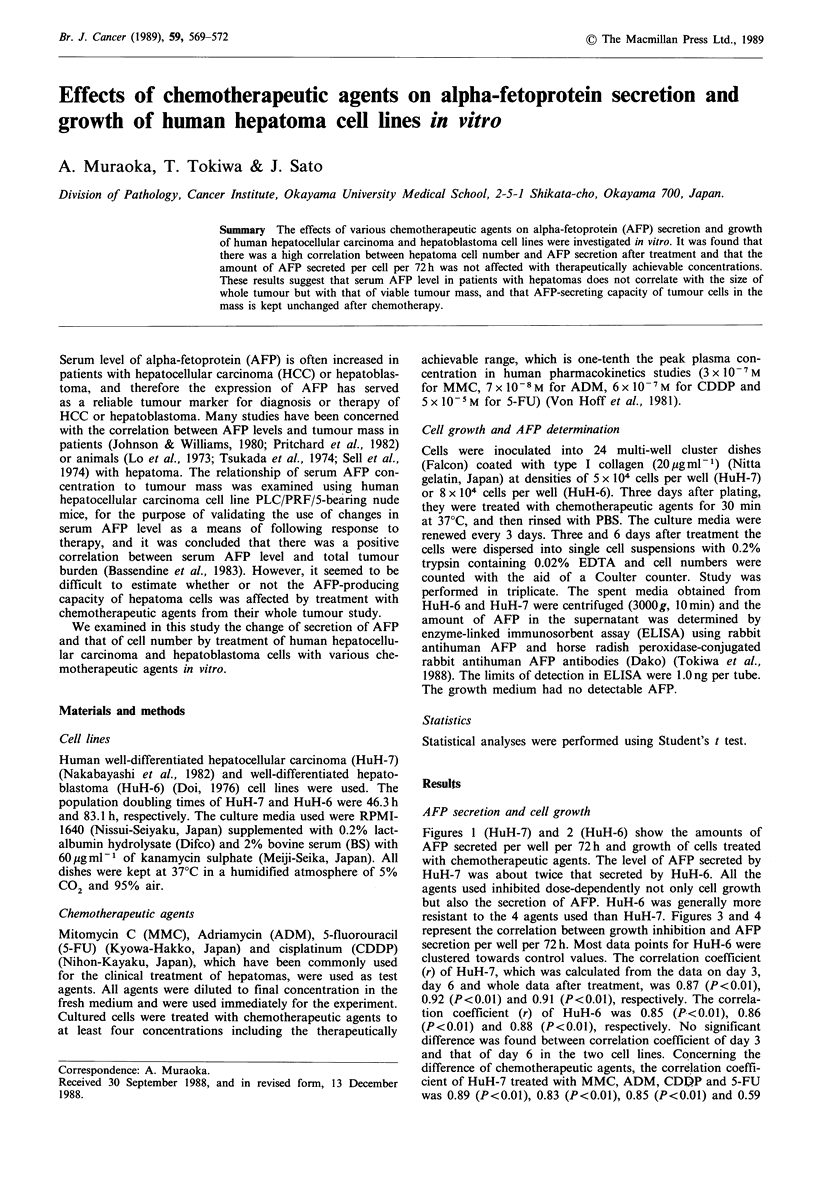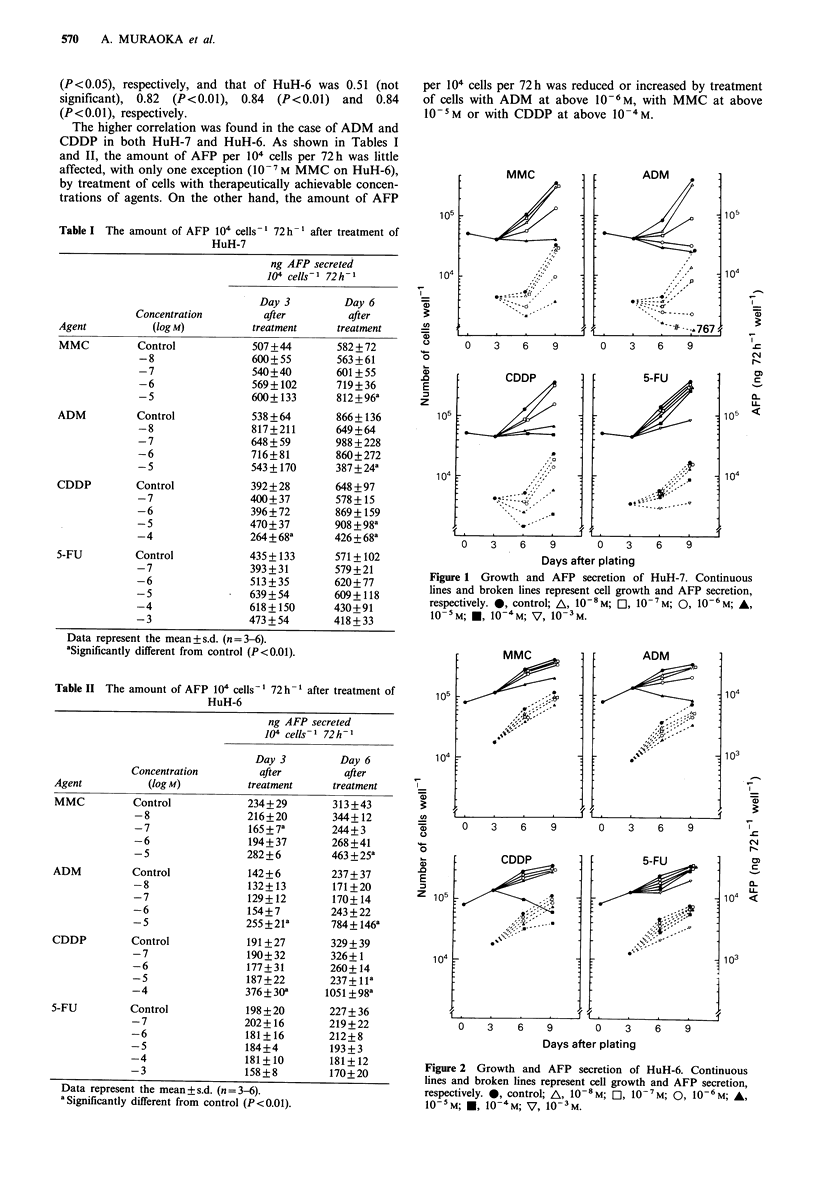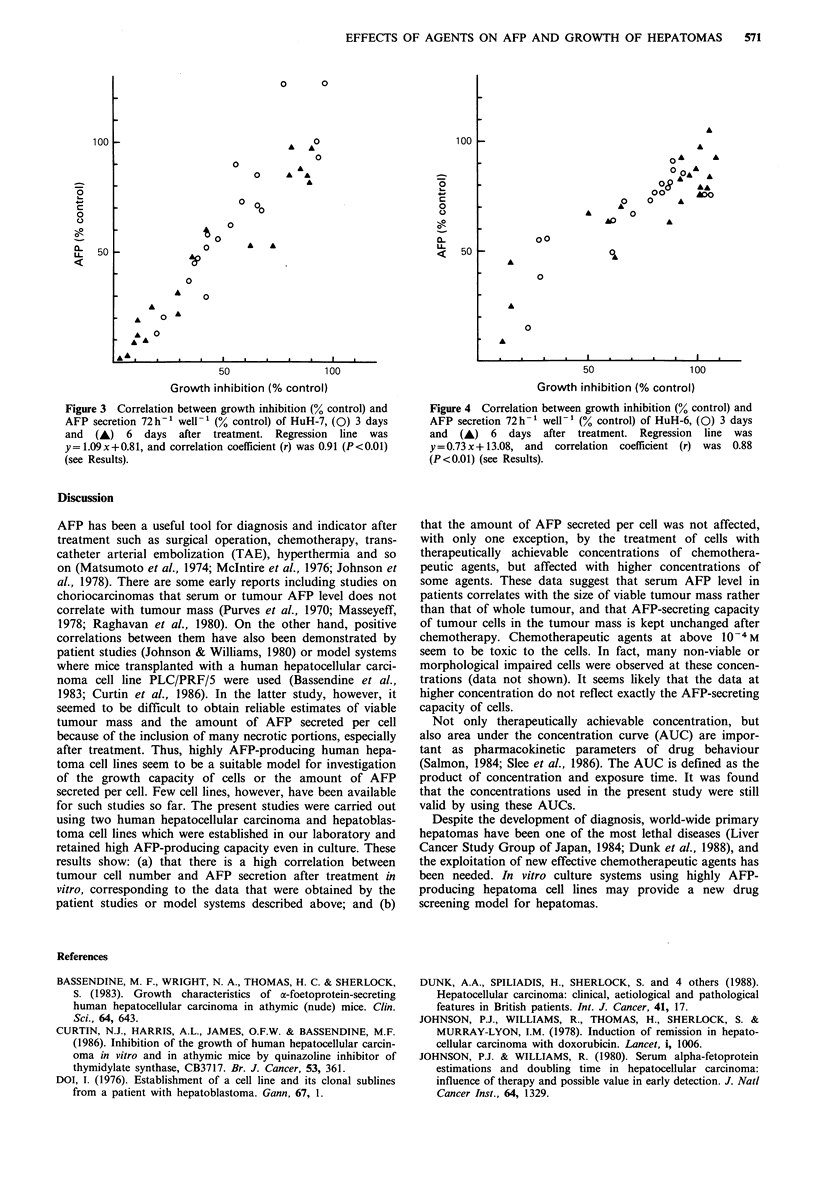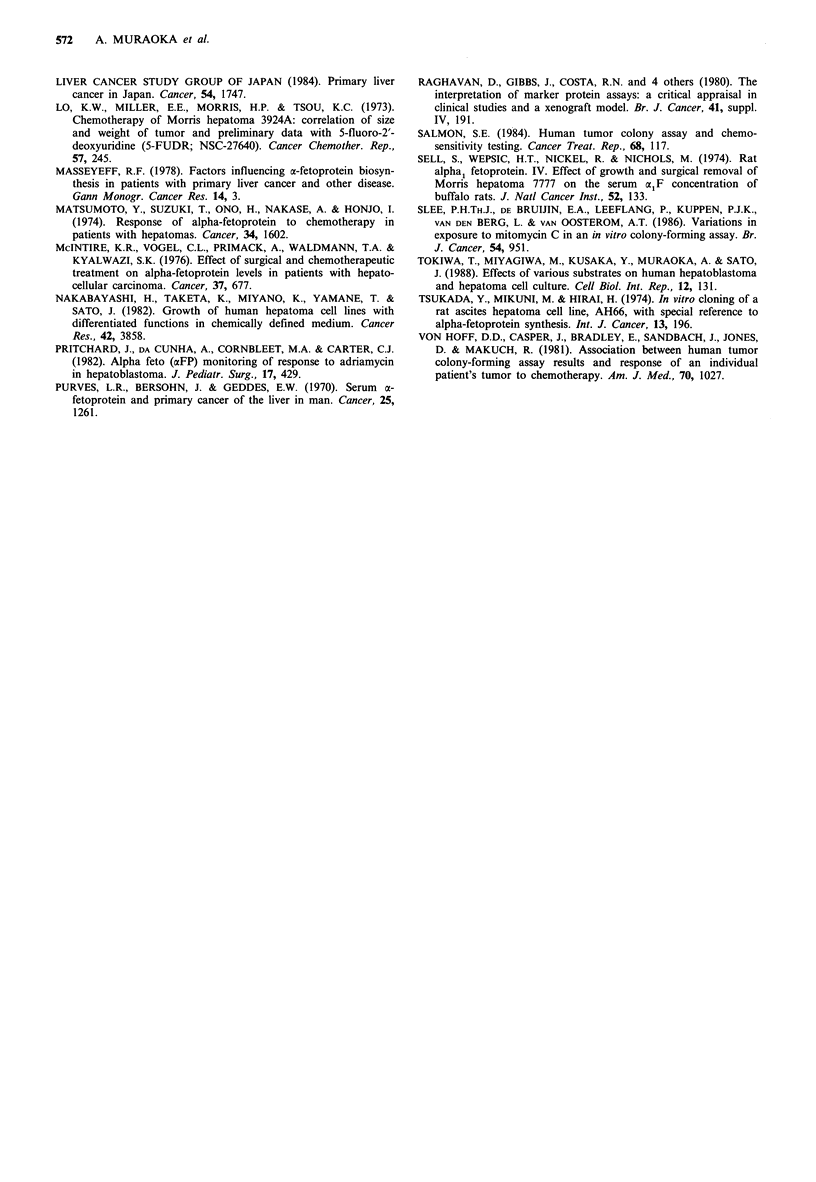# Effects of chemotherapeutic agents on alpha-fetoprotein secretion and growth of human hepatoma cell lines in vitro.

**DOI:** 10.1038/bjc.1989.115

**Published:** 1989-04

**Authors:** A. Muraoka, T. Tokiwa, J. Sato

**Affiliations:** Division of Pathology, Okayama University Medical School, Japan.

## Abstract

The effects of various chemotherapeutic agents on alpha-fetoprotein (AFP) secretion and growth of human hepatocellular carcinoma and hepatoblastoma cell lines were investigated in vitro. It was found that there was a high correlation between hepatoma cell number and AFP secretion after treatment and that the amount of AFP secreted per cell per 72 h was not affected with therapeutically achievable concentrations. These results suggest that serum AFP level in patients with hepatomas does not correlate with the size of whole tumour but with that of viable tumour mass, and that AFP-secreting capacity of tumour cells in the mass is kept unchanged after chemotherapy.


Br. J. Cancer (1989), 59, 569–572 © The Macmillan Press Ltd., 1989

### Effects of chemotherapeutic agents on alpha-fetoprotein secretion and growth of human hepatoma cell lines *in vitro*

A. Muraoka, T. Tokiwa & J. Sato

*Division of Pathology, Cancer Institute, Okayama University Medical School, 2-5-1 Shikata-cho, Okayama 700, Japan.*

**Summary** The effects of various chemotherapeutic agents on alpha-fetoprotein (AFP) secretion and growth of human hepatocellular carcinoma and hepatoblastoma cell lines were investigated *in vitro*. It was found that there was a high correlation between hepatoma cell number and AFP secretion after treatment and that the amount of AFP secreted per cell per 72 h was not affected with therapeutically achievable concentrations. These results suggest that serum AFP level in patients with hepatomas does not correlate with the size of whole tumour but with that of viable tumour mass, and that AFP-secreting capacity of tumour cells in the mass is kept unchanged after chemotherapy.

Serum level of alpha-fetoprotein (AFP) is often increased in patients with hepatocellular carcinoma (HCC) or hepatoblastoma, and therefore the expression of AFP has served as a reliable tumour marker for diagnosis or therapy of HCC or hepatoblastoma. Many studies have been concerned with the correlation between AFP levels and tumour mass in patients (Johnson & Williams, 1980; Pritchard *et al.*, 1982) or animals (Lo *et al.*, 1973; Tsukada *et al.*, 1974; Sell *et al.*, 1974) with hepatoma. The relationship of serum AFP concentration to tumour mass was examined using human hepatocellular carcinoma cell line PLC/PRF/5-bearing nude mice, for the purpose of validating the use of changes in serum AFP level as a means of following response to therapy, and it was concluded that there was a positive correlation between serum AFP level and total tumour burden (Bassendine *et al.*, 1983). However, it seemed to be difficult to estimate whether or not the AFP-producing capacity of hepatoma cells was affected by treatment with chemotherapeutic agents from their whole tumour study.

We examined in this study the change of secretion of AFP and that of cell number by treatment of human hepatocellular carcinoma and hepatoblastoma cells with various chemotherapeutic agents *in vitro*.

### Materials and methods

### Cell lines

Human well-differentiated hepatocellular carcinoma (HuH-7) (Nakabayashi *et al.*, 1982) and well-differentiated hepatoblastoma (HuH-6) (Doi, 1976) cell lines were used. The population doubling times of HuH-7 and HuH-6 were 46.3 h and 83.1 h, respectively. The culture media used were RPMI-1640 (Nissui-Seiyaku, Japan) supplemented with 0.2% lactalbumin hydrolysate (Difco) and 2% bovine serum (BS) with 60 $\mu g\,ml^{-1}$ of kanamycin sulphate (Meiji-Seika, Japan). All dishes were kept at 37°C in a humidified atmosphere of 5% $CO_2$ and 95% air.

### Chemotherapeutic agents

Mitomycin C (MMC), Adriamycin (ADM), 5-fluorouracil (5-FU) (Kyowa-Hakko, Japan) and cisplatinum (CDDP) (Nihon-Kayaku, Japan), which have been commonly used for the clinical treatment of hepatomas, were used as test agents. All agents were diluted to final concentration in the fresh medium and were used immediately for the experiment. Cultured cells were treated with chemotherapeutic agents to at least four concentrations including the therapeutically achievable range, which is one-tenth the peak plasma concentration in human pharmacokinetics studies ($3 \times 10^{-7}$ M for MMC, $7 \times 10^{-8}$ M for ADM, $6 \times 10^{-7}$ M for CDDP and $5 \times 10^{-5}$ M for 5-FU) (Von Hoff *et al.*, 1981).

### Cell growth and AFP determination

Cells were inoculated into 24 multi-well cluster dishes (Falcon) coated with type I collagen (20 $\mu g\,ml^{-1}$) (Nitta gelatin, Japan) at densities of $5 \times 10^4$ cells per well (HuH-7) or $8 \times 10^4$ cells per well (HuH-6). Three days after plating, they were treated with chemotherapeutic agents for 30 min at 37°C, and then rinsed with PBS. The culture media were renewed every 3 days. Three and 6 days after treatment the cells were dispersed into single cell suspensions with 0.2% trypsin containing 0.02% EDTA and cell numbers were counted with the aid of a Coulter counter. Study was performed in triplicate. The spent media obtained from HuH-6 and HuH-7 were centrifuged (3000 *g*, 10 min) and the amount of AFP in the supernatant was determined by enzyme-linked immunosorbent assay (ELISA) using rabbit antihuman AFP and horse radish peroxidase-conjugated rabbit antihuman AFP antibodies (Dako) (Tokiwa *et al.*, 1988). The limits of detection in ELISA were 1.0 ng per tube. The growth medium had no detectable AFP.

### Statistics

Statistical analyses were performed using Student's *t* test.

### Results

### AFP secretion and cell growth

Figures 1 (HuH-7) and 2 (HuH-6) show the amounts of AFP secreted per well per 72 h and growth of cells treated with chemotherapeutic agents. The level of AFP secreted by HuH-7 was about twice that secreted by HuH-6. All the agents used inhibited dose-dependently not only cell growth but also the secretion of AFP. HuH-6 was generally more resistant to the 4 agents used than HuH-7. Figures 3 and 4 represent the correlation between growth inhibition and AFP secretion per well per 72 h. Most data points for HuH-6 were clustered towards control values. The correlation coefficient (*r*) of HuH-7, which was calculated from the data on day 3, day 6 and whole data after treatment, was 0.87 ($P<0.01$), 0.92 ($P<0.01$) and 0.91 ($P<0.01$), respectively. The correlation coefficient (*r*) of HuH-6 was 0.85 ($P<0.01$), 0.86 ($P<0.01$) and 0.88 ($P<0.01$), respectively. No significant difference was found between correlation coefficient of day 3 and that of day 6 in the two cell lines. Concerning the difference of chemotherapeutic agents, the correlation coefficient of HuH-7 treated with MMC, ADM, CDDP and 5-FU was 0.89 ($P<0.01$), 0.83 ($P<0.01$), 0.85 ($P<0.01$) and 0.59

Correspondence: A. Muraoka.
Received 30 September 1988, and in revised form, 13 December 1988.

($P<0.05$), respectively, and that of HuH-6 was 0.51 (not significant), 0.82 ($P<0.01$), 0.84 ($P<0.01$) and 0.84 ($P<0.01$), respectively.

The higher correlation was found in the case of ADM and CDDP in both HuH-7 and HuH-6. As shown in Tables I and II, the amount of AFP per $10^4$ cells per 72 h was little affected, with only one exception ($10^{-7}$ M MMC on HuH-6), by treatment of cells with therapeutically achievable concentrations of agents. On the other hand, the amount of AFP per $10^4$ cells per 72 h was reduced or increased by treatment of cells with ADM at above $10^{-6}$ M, with MMC at above $10^{-5}$ M or with CDDP at above $10^{-4}$ M.

**Table I** The amount of AFP $10^4$ cells$^{-1}$ 72h$^{-1}$ after treatment of HuH-7

| | | *ng AFP secreted $10^4$ cells$^{-1}$ 72 h$^{-1}$* | |
|---|---|---|---|
| *Agent* | *Concentration (log M)* | *Day 3 after treatment* | *Day 6 after treatment* |
| MMC | Control | 507±44 | 582±72 |
| | −8 | 600±55 | 563±61 |
| | −7 | 540±40 | 601±55 |
| | −6 | 569±102 | 719±36 |
| | −5 | 600±133 | 812±96[a] |
| ADM | Control | 538±64 | 866±136 |
| | −8 | 817±211 | 649±64 |
| | −7 | 648±59 | 988±228 |
| | −6 | 716±81 | 860±272 |
| | −5 | 543±170 | 387±24[a] |
| CDDP | Control | 392±28 | 648±97 |
| | −7 | 400±37 | 578±15 |
| | −6 | 396±72 | 869±159 |
| | −5 | 470±37 | 908±98[a] |
| | −4 | 264±68[a] | 426±68[a] |
| 5-FU | Control | 435±133 | 571±102 |
| | −7 | 393±31 | 579±21 |
| | −6 | 513±35 | 620±77 |
| | −5 | 639±54 | 609±118 |
| | −4 | 618±150 | 430±91 |
| | −3 | 473±54 | 418±33 |

Data represent the mean±s.d. ($n$=3–6).
[a]Significantly different from control ($P<0.01$).

**Table II** The amount of AFP $10^4$ cells$^{-1}$ 72h$^{-1}$ after treatment of HuH-6

| | | *ng AFP secreted $10^4$ cells$^{-1}$ 72 h$^{-1}$* | |
|---|---|---|---|
| *Agent* | *Concentration (log M)* | *Day 3 after treatment* | *Day 6 after treatment* |
| MMC | Control | 234±29 | 313±43 |
| | −8 | 216±20 | 344±12 |
| | −7 | 165±7[a] | 244±3 |
| | −6 | 194±37 | 268±41 |
| | −5 | 282±6 | 463±25[a] |
| ADM | Control | 142±6 | 237±37 |
| | −8 | 132±13 | 171±20 |
| | −7 | 129±12 | 170±14 |
| | −6 | 154±7 | 243±22 |
| | −5 | 255±21[a] | 784±146[a] |
| CDDP | Control | 191±27 | 329±39 |
| | −7 | 190±32 | 326±1 |
| | −6 | 177±31 | 260±14 |
| | −5 | 187±22 | 237±11[a] |
| | −4 | 376±30[a] | 1051±98[a] |
| 5-FU | Control | 198±20 | 227±36 |
| | −7 | 202±16 | 219±22 |
| | −6 | 181±16 | 212±8 |
| | −5 | 184±4 | 193±3 |
| | −4 | 181±10 | 181±12 |
| | −3 | 158±8 | 170±20 |

Data represent the mean±s.d. ($n$=3–6).
[a]Significantly different from control ($P<0.01$).

**Figure 1** Growth and AFP secretion of HuH-7. Continuous lines and broken lines represent cell growth and AFP secretion, respectively. ●, control; △, $10^{-8}$ M; □, $10^{-7}$ M; ○, $10^{-6}$ M; ▲, $10^{-5}$ M; ■, $10^{-4}$ M; ▽, $10^{-3}$ M.

**Figure 2** Growth and AFP secretion of HuH-6. Continuous lines and broken lines represent cell growth and AFP secretion, respectively. ●, control; △, $10^{-8}$ M; □, $10^{-7}$ M; ○, $10^{-6}$ M; ▲, $10^{-5}$ M; ■, $10^{-4}$ M; ▽, $10^{-3}$ M.

**Figure 3** Correlation between growth inhibition (% control) and AFP secretion 72h$^{-1}$ well$^{-1}$ (% control) of HuH-7, (○) 3 days and (▲) 6 days after treatment. Regression line was $y = 1.09x + 0.81$, and correlation coefficient ($r$) was 0.91 ($P < 0.01$) (see Results).

**Figure 4** Correlation between growth inhibition (% control) and AFP secretion 72h$^{-1}$ well$^{-1}$ (% control) of HuH-6, (○) 3 days and (▲) 6 days after treatment. Regression line was $y = 0.73x + 13.08$, and correlation coefficient ($r$) was 0.88 ($P < 0.01$) (see Results).

### Discussion

AFP has been a useful tool for diagnosis and indicator after treatment such as surgical operation, chemotherapy, transcatheter arterial embolization (TAE), hyperthermia and so on (Matsumoto *et al.*, 1974; McIntire *et al.*, 1976; Johnson *et al.*, 1978). There are some early reports including studies on choriocarcinomas that serum or tumour AFP level does not correlate with tumour mass (Purves *et al.*, 1970; Masseyeff, 1978; Raghavan *et al.*, 1980). On the other hand, positive correlations between them have also been demonstrated by patient studies (Johnson & Williams, 1980) or model systems where mice transplanted with a human hepatocellular carcinoma cell line PLC/PRF/5 were used (Bassendine *et al.*, 1983; Curtin *et al.*, 1986). In the latter study, however, it seemed to be difficult to obtain reliable estimates of viable tumour mass and the amount of AFP secreted per cell because of the inclusion of many necrotic portions, especially after treatment. Thus, highly AFP-producing human hepatoma cell lines seem to be a suitable model for investigation of the growth capacity of cells or the amount of AFP secreted per cell. Few cell lines, however, have been available for such studies so far. The present studies were carried out using two human hepatocellular carcinoma and hepatoblastoma cell lines which were established in our laboratory and retained high AFP-producing capacity even in culture. These results show: (a) that there is a high correlation between tumour cell number and AFP secretion after treatment *in vitro*, corresponding to the data that were obtained by the patient studies or model systems described above; and (b) that the amount of AFP secreted per cell was not affected, with only one exception, by the treatment of cells with therapeutically achievable concentrations of chemotherapeutic agents, but affected with higher concentrations of some agents. These data suggest that serum AFP level in patients correlates with the size of viable tumour mass rather than that of whole tumour, and that AFP-secreting capacity of tumour cells in the tumour mass is kept unchanged after chemotherapy. Chemotherapeutic agents at above $10^{-4}$ M seem to be toxic to the cells. In fact, many non-viable or morphological impaired cells were observed at these concentrations (data not shown). It seems likely that the data at higher concentration do not reflect exactly the AFP-secreting capacity of cells.

Not only therapeutically achievable concentration, but also area under the concentration curve (AUC) are important as pharmacokinetic parameters of drug behaviour (Salmon, 1984; Slee *et al.*, 1986). The AUC is defined as the product of concentration and exposure time. It was found that the concentrations used in the present study were still valid by using these AUCs.

Despite the development of diagnosis, world-wide primary hepatomas have been one of the most lethal diseases (Liver Cancer Study Group of Japan, 1984; Dunk *et al.*, 1988), and the exploitation of new effective chemotherapeutic agents has been needed. *In vitro* culture systems using highly AFP-producing hepatoma cell lines may provide a new drug screening model for hepatomas.

### References

BASSENDINE, M. F., WRIGHT, N. A., THOMAS, H. C. & SHERLOCK, S. (1983). Growth characteristics of α-foetoprotein-secreting human hepatocellular carcinoma in athymic (nude) mice. *Clin. Sci.*, **64**, 643.

CURTIN, N.J., HARRIS, A.L., JAMES, O.F.W. & BASSENDINE, M.F. (1986). Inhibition of the growth of human hepatocellular carcinoma *in vitro* and in athymic mice by quinazoline inhibitor of thymidylate synthase, CB3717. *Br. J. Cancer*, **53**, 361.

DOI, I. (1976). Establishment of a cell line and its clonal sublines from a patient with hepatoblastoma. *Gann*, **67**, 1.

DUNK, A.A., SPILIADIS, H., SHERLOCK, S. and 4 others (1988). Hepatocellular carcinoma: clinical, aetiological and pathological features in British patients. *Int. J. Cancer*, **41**, 17.

JOHNSON, P.J., WILLIAMS, R., THOMAS, H., SHERLOCK, S. & MURRAY-LYON, I.M. (1978). Induction of remission in hepatocellular carcinoma with doxorubicin. *Lancet*, **i**, 1006.

JOHNSON, P.J. & WILLIAMS, R. (1980). Serum alpha-fetoprotein estimations and doubling time in hepatocellular carcinoma: influence of therapy and possible value in early detection. *J. Natl Cancer Inst.*, **64**, 1329.

LIVER CANCER STUDY GROUP OF JAPAN (1984). Primary liver cancer in Japan. *Cancer*, **54**, 1747.

LO, K.W., MILLER, E.E., MORRIS, H.P. & TSOU, K.C. (1973). Chemotherapy of Morris hepatoma 3924A: correlation of size and weight of tumor and preliminary data with 5-fluoro-2'-deoxyuridine (5-FUDR; NSC-27640). *Cancer Chemother. Rep.*, **57**, 245.

MASSEYEFF, R.F. (1978). Factors influencing α-fetoprotein biosynthesis in patients with primary liver cancer and other disease. *Gann Monogr. Cancer Res.* **14**, 3.

MATSUMOTO, Y., SUZUKI, T., ONO, H., NAKASE, A. & HONJO, I. (1974). Response of alpha-fetoprotein to chemotherapy in patients with hepatomas. *Cancer*, **34**, 1602.

McINTIRE, K.R., VOGEL, C.L., PRIMACK, A., WALDMANN, T.A. & KYALWAZI, S.K. (1976). Effect of surgical and chemotherapeutic treatment on alpha-fetoprotein levels in patients with hepatocellular carcinoma. *Cancer*, **37**, 677.

NAKABAYASHI, H., TAKETA, K., MIYANO, K., YAMANE, T. & SATO, J. (1982). Growth of human hepatoma cell lines with differentiated functions in chemically defined medium. *Cancer Res.*, **42**, 3858.

PRITCHARD, J., DA CUNHA, A., CORNBLEET, M.A. & CARTER, C.J. (1982). Alpha feto (αFP) monitoring of response to adriamycin in hepatoblastoma. *J. Pediatr. Surg.*, **17**, 429.

PURVES, L.R., BERSOHN, J. & GEDDES, E.W. (1970). Serum α-fetoprotein and primary cancer of the liver in man. *Cancer*, **25**, 1261.

RAGHAVAN, D., GIBBS, J., COSTA, R.N. and 4 others (1980). The interpretation of marker protein assays: a critical appraisal in clinical studies and a xenograft model. *Br. J. Cancer*, **41**, suppl. IV, 191.

SALMON, S.E. (1984). Human tumor colony assay and chemosensitivity testing. *Cancer Treat. Rep.*, **68**, 117.

SELL, S., WEPSIC, H.T., NICKEL, R. & NICHOLS, M. (1974). Rat $alpha_1$ fetoprotein. IV. Effect of growth and surgical removal of Morris hepatoma 7777 on the serum $\alpha_1$F concentration of buffalo rats. *J. Natl Cancer Inst.*, **52**, 133.

SLEE, P.H.TH.J., DE BRUIJIN, E.A., LEEFLANG, P., KUPPEN, P.J.K., VAN DEN BERG, L. & VAN OOSTEROM, A.T. (1986). Variations in exposure to mitomycin C in an *in vitro* colony-forming assay. *Br. J. Cancer*, **54**, 951.

TOKIWA, T., MIYAGIWA, M., KUSAKA, Y., MURAOKA, A. & SATO, J. (1988). Effects of various substrates on human hepatoblastoma and hepatoma cell culture. *Cell Biol. Int. Rep.*, **12**, 131.

TSUKADA, Y., MIKUNI, M. & HIRAI, H. (1974). *In vitro* cloning of a rat ascites hepatoma cell line, AH66, with special reference to alpha-fetoprotein synthesis. *Int. J. Cancer*, **13**, 196.

VON HOFF, D.D., CASPER, J., BRADLEY, E., SANDBACH, J., JONES, D. & MAKUCH, R. (1981). Association between human tumor colony-forming assay results and response of an individual patient's tumor to chemotherapy. *Am. J. Med.*, **70**, 1027.